# Remote Learning in Transnational Education: Relationship between Virtual Learning Engagement and Student Academic Performance in BSc Pharmaceutical Biotechnology

**DOI:** 10.3390/pharmacy10010004

**Published:** 2021-12-27

**Authors:** Taher Hatahet, Ahmed A.Raouf Mohamed, Maryam Malekigorji, Emma K. Kerry

**Affiliations:** 1 School of Pharmacy, Queens University Belfast, Belfast BT9 7BL, UK; m.malekigorji@qub.ac.uk (M.M.); E.Kerry@qub.ac.uk (E.K.K.); 2 Queen’s University Joint College, China Medical University, Shenyang 110122, China; 3 School of Electronics, Electrical Engineering and Computer Science, Queens University Belfast, Belfast BT9 5AG, UK; amohamed06@qub.ac.uk

**Keywords:** academic performance, linear regression, modelling, optimization, student engagement, transnational education, virtual learning environment

## Abstract

The 21st century has seen dramatic changes to education delivery which have widened the scope of transnational education and remote learning via various virtual learning environments (VLEs). Efficient remote teaching activities require students to be engaged with taught materials and academic staff, and for educators to be able to track and improve student engagement. This article describes the generation of a predictive mathematical model for students’ exam performance using VLE engagement indicators and coursework marks together to enable the creation of a model with a correlation coefficient of 0.724. This article examines the relationship of each of these variables with final exam marks, as well as the addition of personal related variable X on the generated model’s accuracy. The generated models show that each variable had a different impact on the prediction of the final exam mark. The results’ analysis suggests that coursework marks and total VLE page views were the major attributes, while personal factors were also found to greatly impact model accuracy. Considering the case of outliers, who were students with low VLE engagement achieving high exam marks, it is proposed that personal factors, such as behavioural factors and study style, also have a significant effect on student academic attainment. The generated model can be used by students to improve self-efficacy by adjusting their study style and by educators to provide early interventions to support disengaged students. This model can be replicated in different remote learning settings and transnational education, and the findings might be insightful for courses with remote learning strategies to investigate the key educational, personal and engagement parameters for students’ overall success.

## 1. Introduction

Technology has been increasingly used and applied in many aspects of science. The new era of the digitalised world has also affected higher education. As social media have developed, virtual learning environments (VLEs) have been designed to create an online space for student learning and development. VLEs enable students to access learning resources remotely, such as lecture notes, videos, or quizzes for self-assessment to test learner’s understanding. They also provide discussion forums to enable asynchronous interactions between peers and teachers [1]. The online teaching and learning environment has not only changed the student learning experience, but has also facilitated teaching for educators. Further, it has opened the door for transnational education to evolve. High-ranking universities with campuses in different geographic locations now rely on VLE technology to deliver teaching remotely [2]. The VLE infrastructure is not limited to the teacher student interface. Analytics of student interaction with the VLE content can now be used to indirectly track and follow a student’s learning experience [3].

VLEs with multiple functionalities and tools have been used to monitor student engagement and interaction with content and different learning resources. According to Kuh, “*Student Engagement is the extent to which (students) take part in educationally effective practices*” [4]. In addition, the National Survey of Student Engagement was established in 1999 to reflect on the role of engagement in student learning and development [5]. Student engagement has been shown to be a key parameter for better learning experiences and higher academic attainment [6,7]. Furthermore, engagement has been shown to be even more important when part or all of a degree is delivered remotely. However, remote teaching is a dichotomy of the opportunities that come with the endless capabilities of the online teaching environment and the disadvantages that come with the learning process being reliant on student self-esteem, motivation and engagement [8]. Although students studying at distance in transnational education are offered a range of remote activities that enable student self-assessment [9], further support is needed to help students understand their own performance compared to a more defined success scale set up by educators themselves [10]. Therefore, if poorly engaging students can be identified before final summative assessments, educators would then be able to take an essential preventive approach to support students [10].

Canvas is an example of a top-ranked VLE and is used by Queen’s University Belfast (QUB) in the delivery of both undergraduate and postgraduate teaching [11]. Canvas provides a friendly learning environment for both teachers and students. It enables educators to merge videos, voice recordings, written texts, images, quizzes, and discussions in any educationally suitable manner they wish. Lectures can be structured as webpages with multiple sorts of information and these pages can be linked to quizzes or discussions [12]. For students, Canvas gathers deadlines in a comprehensive calendar, sends out submission reminders and links students to educators by sending pop up notifications to their smartphone application each time a lecturer posts a new announcement to Canvas [13]. Canvas also has several tools in the course analytics section that can indirectly reflect on student engagement with the taught materials. The appropriate use of these tools can help educators generate a progress scale, which students can use to assess their own performance and educators can use to identify at-risk students.

Canvas VLE engagement indicators are mainly the total number of page views, the last page view, which reflects the student’s last page view, and the total number of participations. VLE engagement indicators alongside coursework marks collected throughout the year can be a useful predictive tool for future student exam performance. However, each of these indicators cannot be used alone in making decisions on students’ engagement or academic performance. Linking these indicators with coursework marks in a comprehensive model with input and output data can take advantage of each indicator, giving each indicator its appropriate weight and appreciation. Creating a predictive model would help educators perform preventive actions for students at risk of failure due to disengagement or low coursework marks. In parallel, publishing the generated model for students would help them to correctly adjust their own progress based on their self-assessment which becomes better calibrated with time [8].

This project aims to better understand the relationship among online engagement indicators, continuous assessment marks and personal factors in predicting student performance in final exams. It is hoped that the results will help academic staff teaching in online transnational education to provide timely support for poorly engaged students before taking the final examination, so that the failure rate can be reduced, and a better learning experience can be provided to all students. The broader purpose of this is to see whether coursework marks and VLE engagement indicators can be used by students for more effective self-assessment during the course of their degrees.

## 2. Materials and Methods

### 2.1. Study Participants and Studied Course

The generated VLE engagement report, as well as the examination and coursework scores, were collected for 55 students who were enrolled within Level 2 of the BSc Pharmaceutical Biotechnology degrees for the academic year 2020–2021 off-campus in Shenyang, People’s Republic of China, as part of the transnational education programme run by QUB in partnership with China Medical University. The students’ scores used in this study are illustrated in Supplementary data Figures S1 and S2.

The students’ scores were collected from Level 2 Pharmaceutical Formulation module. The course focusses on formulation principles for different products, such as oral dosage forms, suspensions, solutions, etc. The course falls within the pharmaceutical technology branch of science. The course is taught as a hybrid model of 28 pre-recorded lectures and 7 live recap sessions delivered using online MS Teams platforms (Microsoft, Redmond, WA, USA). The course has also 9 practical classes, 1 oral presentation, 5 self-study sessions and 2 class tests. The course has quizzes embedded within the pre-recorded lectures and a discussion board per taught topic (can be between 2 and 5 lectures). Students are requested to watch content following the timetable and prepare the quizzes and discussions before attending live sessions. The course has further readings associated with each topic that are outside the VLE environment, such as articles, textbooks, and news reports.

### 2.2. Study Design

**Figure 1:**
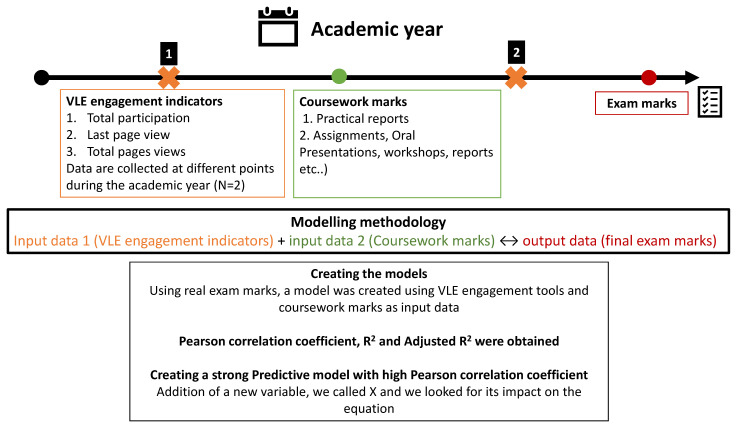
A schematic representation of the study design.

Mathematical models were created using the Canvas VLE engagement indicators. Student coursework marks throughout the year were used as input data and the students’ final exam mark was used as an output for each student. The generated models were compared in terms of correlation coefficients and statistical significance to assess whether they could be used as a predictive tool to identify future students who were at risk of failure at an early stage of the course and before taking their final examinations (Figure 1).

The first set of input data was a continuous collection of students VLE engagement indicators. Canvas provides several indicators of interaction with the VLE for each user ID (student). These indicators are not directly linked to a specific teaching or learning content but rather a holistic interaction with the VLE. Canvas generates a personalized report that indicates (i) total participation, (ii) last page view and (iii) total page views. The total participation indicator shows how many times a student actively engaged with an online activity such as submitting an assignment, commenting on a discussion board, or attempting a quiz. The last page view is equivalent to the student’s last login date showing whether student engagement with the content has been continuous. The total page views indicator can be regarded as an indirect indicator of time spent by each student on the VLE. These three indicators were collected twice for all students and were used as VLE engagement variables.

The second set of input data was gathered from the coursework continuous assessment marks that students undertook during the teaching term, such as practical reports, assignments, and oral presentations, which collectively contribute to the final course mark.

The output data used to create the mathematical models was the final examination mark of each student. The models were created using real data and the Pearson correlation coefficient, R^2^ and adjusted R^2^ were obtained and assessed for strength of correlation and validity of the generated models. The associated weights of each of these variables (i.e., individual input data sets in the generated equations) were used to investigate the importance of each variable on student success in final examinations.

### 2.3. Data Modelling and Analysis

**Figure 2:**
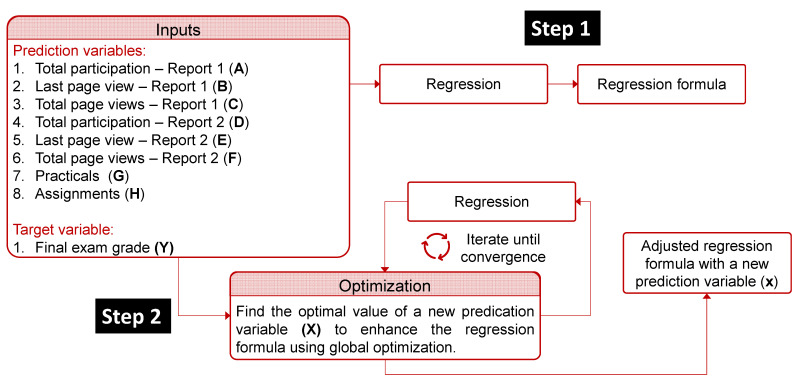
A schematic representation of the stages of the modelling process.

The modelling process is divided into two steps as outlined in Figure 2 using MATLAB R2020b (The MathWorks Inc., Natick, MA, USA) [14]. The prediction variables are denoted by letters (from *A* to *H*). The exam mark as an output variable is symbolized by *Y*. The first step of the modelling, illustrated in Figure 2, was to produce a simple linear regression model between the predictors and the output. During this step, two regression models were generated. The first model is a relationship between all the predictors and the output, while the second model is a relationship between the best combination with the least number of predictors and the output. In the next step, illustrated in Figure 2, we aimed to identify the optimal value of an additional variable (symbolized by *X*) that could be added to improve the regression model’s accuracy. This was formulated as black-box optimization [15], then solved using the Genetic Algorithm (GA) [16] due to its capability of providing good solutions in a reasonable time. The objective function of this optimization problem is to maximize the Pearson correlation coefficient (R-value) using a decision variable *X* that has a lower bound of 0 and an upper bound of 10. The GA optimizer iterates by updating the decision variables until convergence, which represents an optimal value of the Pearson correlation coefficient (R = 1) for each student. The outputs of this step (Step 2) were two other regression models.

Afterwards, the outliers were detected and discarded using the generalized extreme studentized deviate test for outliers [17]. This iterative method removes one outlier per iteration based on hypothesis testing. This method assumes that the data is normally distributed and has the capability to perform well when there are multiple outliers masking each other. The previous steps (Step 1 and Step 2) are then repeated without outliers. Therefore, a total number of eight regression models were developed with statistical significance when *p* < 0.05 donated by *, *p* < 0.01 donated by ** and *p* <0.001 donated by ***.

### 2.4. Data Storage and Accessibility

Data were stored on password encrypted hardware that was only accessible for the authors of this article. The linkage between students input and output data were anonymized using an arbitrary ID during the analysis. The excel sheet that was used to perform the task was only accessible to the corresponding author and was permanently deleted after the task.

## 3. Results

### 3.1. Effect of Outliers on Individual Indicators Relationship to Exam Marks

**Figure 3:**
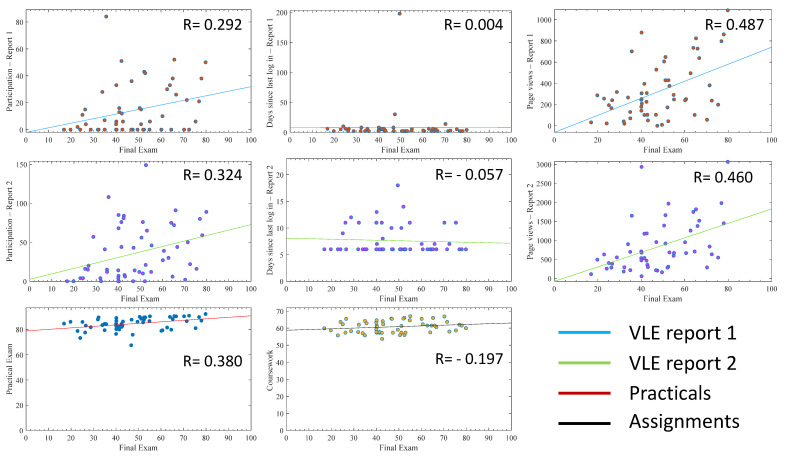
The relationship of individual variables with the final exam mark plotted as scatter with a least-square line including all students (with outliers). The *y*-axis represents the value of the indicator (total participation number, number of days since last login and total number of page views) or the coursework and practical marks per student (*x*-axis). The R-value is the Pearson correlation coefficient. Graphs are plotted with the final exam mark on the *x*-axis and each variable on the *y*-axis. VLE stands for virtual learning environment.

Figure 3 presents the individual relationship of each variable to the student exam mark including all students (with outliers). It was observed that the Pearson correlation coefficients (R-value) coming from VLE report 2 variables were better than the data obtained in VLE report 1, which was generated early in the academic year in terms of the total participation (*A* vs. *D*) and last page view (*B* vs. *E*). However, both parameters alone did not show a strong correlation to exam marks. Higher R-values were observed with total page views than with other parameters without changing in terms of correlation to the exam mark over the course of the year. In the same manner, practicals and assignments alone failed to show strong relationships to the final exam mark with R values of less than 0.4.

**Figure 4:**
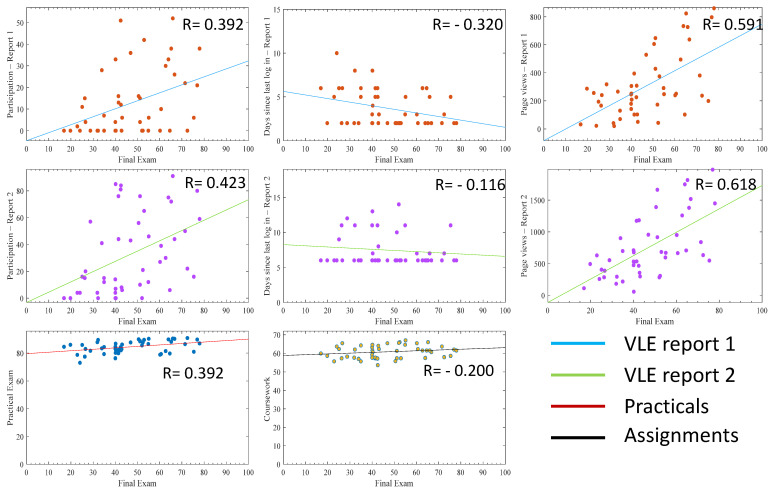
The relationship of individual variables with the final exam mark plotted as scatter with a least-square line including all students (without outliers). The *y*-axis represents the value of the indicator (total participations number, number of days since last log in, total number of pages views) or the coursework and practicals marks per student (*x*-axis). The R-value is the Pearson correlation coefficient. Graphs are plotted with the final exam mark on the *x*-axis and each variable on the *y*-axis. VLE stands for virtual learning environment.

By removing the outliers from the models (Figure 4), all the correlation coefficients generated from VLE engagement indicators improved. This increase was not apparent in variables *H* and *G*, related to coursework marks. With regards to the R-values, variables *D* (total participation) and *F* (total page views) in VLE report 2 were the highest and were selected for modelling with a reduced number of variables in the model (Section 3.3). Variables *G* (practicals) and *H* (assignments) were much more stable to outliers and were also selected for reduced number of variable modelling (Section 3.3). It is worth mentioning that the outliers were identified to be eight students (Section 2.4).

### 3.2. Modelling Using All Variables

When all variables were used in the creation of the model in a simple linear regression model, Equation (1) was obtained with a correlation coefficient of 0.6 (Table 1 and Table 2). With regards to the weight of each variable in Equation (1), the *H* and *G* (coursework marks) variables are the main contributors to the equation. Last page view (*E*) extracted from the VLE engagement report 2 also has a higher contribution to the model than other VLE engagement indicators. The generated model was statistically significant with a *p*-value < 0.01 (Table 2). In relation to optimisation of the equation with a correlation coefficient of 1, the optimisation step was carried out with a new predictor (adjusting variable X) in Equation (2) (Table 1). Equation (2) reinforced the remark that variables *H* and *G* (coursework marks) are high contributors in predicting final exam marks. From the VLE engagement point of view, the variable *E* (Last page view) from report 2 seemed to be crucial as well as an indicator with its high weighting reflecting a student’s overall engagement with the VLE having also accessed the VLE late in term. Equation (2) highlights the huge impact of the personal variable *X* on *Y* (exam marks), where this variable *X* could be more of a personal factor of behavioural origin amongst students. The *X* optimised generated model was statistically significant with *p*-value < 0.001 and a perfect fit (R^2^ = 1) (Table 2). The outcome of removing outliers was clear on all variables modelling with the Pearson correlation coefficient increased from 0.6 in Equation (1) to 0.724 in Equation (3) (Table 2). Removing the outliers improved the overall correlations without changing the importance of variables *G*, *H* and *E* for the equations.

### 3.3. Modelling Using E, F, G and H Variables

Because VLE engagement report 1 variables showed low weight in the generated models, these variables were removed from consideration and new models were created using four variables instead of eight (Table 1, Equations (5)–(8)). The use of fewer variables affected the overall Pearson correlation coefficients to 0.585 with outliers and to 0.7 without outliers (Table 2, Equations (5) and (7)). Again, the removal of outliers improved the correlation and the addition of an *X* variable showed to predominate in top match equations (Table 1 and Table 2, Equations (6) and (8)).

The results of the proposed prediction models for the final exam marks versus the actual exam marks are illustrated in Supplementary Data Figure S3.

## 4. Discussion

The advances of digital learning in higher education has resulted in students worldwide having greater accessibility to learning resources and has opened new frontiers for transnational education [18]. The majority of universities are now using VLEs to support the delivery of their courses. These VLEs extend the learning experience outside the university and provide a 24/7 platform for students to engage with learning [19]. Tracking student engagement is crucial for both educators and learners, where educators can track students’ progress and provide timely support and where learners can develop self-efficacy and adjust their learning style when needed [20]. This was clearly evident during the COVID-19 pandemic with the necessary move to online education [21]. The ability to create a predictive model using numerical tools will have a great impact on students and staff, where students can use such a model as a self-assessment tool to help them realise their need to adjust their learning strategies to improve their academic performance [9]. From a teaching point of view, having a predictive model will help educators to take preventive rather than corrective actions by contacting poorly engaged students on the course at an early stage of their studies and prior to summative assessments [22].

The VLE indirectly provides a way of tracking student’s engagement with taught material in a numerical way. These VLE engagement indicators can be collected from course analytics and include total participation, last page view and total page views. The total participation indicates how often students submit an assignment, ask or answer questions on a discussion board, or attempt an online quiz. Last page view means the last date when students accessed the VLE and total page views shows how many pages of the course have been viewed by students, where more page views are likely to indicate longer studying times and more engagement. Being able to collect this information several times during the academic year enables the progressive follow up where more accurate predictions can be generated towards exam time. To further improve the designed model, directly related variables were considered and were found to be the coursework marks submitted by students during the year. These marks are part of the final mark; thus, they are seriously considered by students.

By analysing each of these variables separately for all students, correlation coefficients below 0.5 were measured with the highest number of total page views (Figure 3). The VLE variables collected early in the year (report 1) showed lower correlation coefficients than the ones collected at a later stage of the year (report 2) (Figure 1 and Figure 2). This shows that VLE variables become more coherent and robust with time, not forgetting that data collected in report 2 inherently contain the data in report 1 as data collection is cumulative. The last page view confirmed in report 2 revealed a negative correlation with final marks, which is coherent with the reality that students who did not log into the VLE for a long time before their final examinations were certainly less engaged and had lower academic performance (Figure 1 and Figure 2) [23]. The removal of outliers increased the correlation coefficients in all variables related to the VLE, reaching up to 0.618 with total page views, which indicates a student studying style impacting on the correlation when using all students, as some students may engage less with the VLE but perform quite well in examinations (Figure 4). This observation was also reported elsewhere, where demographic and behavioural features impact student VLE engagement style [23,24]. The use of all of these variables in a simple linear regression model generated a correlation coefficient of 0.6 (Equation (1)) and, by removing the outliers, this increased to 0.724 (Equation (3)) (Table 2). The use of these variables enabled a high score of correlation coefficient compared to other studies that only used total page views (0.35) [25] and (0.299) [26]. A correlation coefficient of 0.724 makes possible group predictions that are accurate enough for the prediction of students’ exam marks [27].

The coursework marks’ relationship with the final exam marks was less affected by outliers, again supporting that some students may not be engaging with a VLE but may be still performing well in coursework that contributes to the final mark (Figure 3 and Figure 4). Even though the simple individual relationships yielded lower correlation coefficients than total page views of the VLE (0.392 and 0.2 vs. 0.681), their contribution to the overall model seemed to be high in all generated equations with or without outliers (Table 1). Although the correlation of last page view (*E*) was negative with respect to the final mark (*Y*), its existence improved the model accuracy by 5%. This is because it contributed to capturing the model uncertainties when it was accompanied by other predictors (i.e., total page views (*F*), practical reports (*G*) and assignments (*H*)) (Table 2). Similarly, the correlation coefficient of assignments (*H*) was low; however, its removal worsened the model accuracy by 13%. In addition, the assignments (*H*) and practical reports (*G*) proved to be essential and could not be discarded in building such a predictive model. On the other hand, removing the outliers showed to improve the predictive model accuracy by 20% on average (Table 2).

With the aim of getting an equation with a correlation coefficient of 1, an *X* variable was added to the model, assuming that this *X* variable covered the demographic factors, behavioural factors, personal factors, and the learning style of the students. In all the generated equations, *X* seemed to be the largest contributor to the equations (Table 1). The *X* variable seemed to be more important than all other variables that were included in this study. This *X* variable can be a substitute for other variables not detected in this study, for example, the total number of hours studied or students’ intelligence [27]. The high impact of the *X* variable on the productivity of the generated model, along with the observed outliers whose use of the VLE does not correlate with their exam performance, highlights the presence of different learner types that, according to Lee, can be categorized to model students, traditionalists, geeks and the disengaged [28]. Then, the possible intervention could be towards the disengaged students, to make sure they are moving well in the course and to provide them with timely support.

The generated equations highlighted the minimal impact of the VLE report 1 variable on final exam marks such that, by removing them and simplifying report 2 variables to total page views only, reduced the correlation coefficient from 0.724 in Equation (3) to 0.7 in Equation (7); therefore, the model accuracy would be worsened by only 3%. (Table 2). However, this model is simpler and can be used by staff at a late stage of the academic year with a root mean squared error of 12.4%. In the UK, the classification system for third, lower second, upper second and first class is in the range of 10%, i.e., 40–50%, 50–60%, 60–70% and above 70%. This means this model is able to predict a change in exam marks representative of a change in degree classification [29]. The full model with all variables included can be published early in the year to all students, so that they can use it to adjust their learning styles if their initial assignments’ marks are not satisfactory. In the 2021–2022 academic year, the model is guiding us with student support meetings for students that had low scores in the main parameters investigated in the full model.

Further understanding of the *X* variable is needed to be able to complete the image of the main contributions of high academic achievements for students studying remotely in transnational education. The *X* variable can be a mix of variables related to society, culture, politics and economy, for example, students who do work and have minimal VLE interactions, or who are traditionalists, according to Lee’s model [28]. *X* can also be impacted by student’s motivation and cognitive abilities [30]. Finally, of huge importance is the actual impact of the VLE surveillance on learners and their styles [31].

The work mainly focused on improving predictive modelling of VLE engagement tools and was limited to one taught subject, not to the whole year of study subjects. As a result, caution should be taken not to overtake the same model to other subjects. Modelling of other subjects should be conducted in parallel in case the overall performance is envisaged. We did not interview students or seek their perceptions to further comprehend the meanings of the *X* variable. The models are also cohort-specific and again caution needs to be taken not to use but rather reproduce in other cohorts of students. Future work is required to survey students on the main attributes of the *X* variable and its role in engagement with the VLE. Extension of this work can be towards applying predictive modelling for full semester subjects in order to capture weak overall performers.

## 5. Conclusions

This article describes the process of generating a predictive mathematical model regarding student exam performance using virtual learning environment engagement indicators (VLE reports) collected at two different time points during the academic year and coursework marks attained by students. The generated models tested for the effect of outliers, the addition of an *X* variable representing student learning styles and the inclusion of all variables or selected ones. The enhanced model without outliers enabled an accurate group prediction of students’ final exam mark with a margin of error of one-degree classification. Coursework marks and total page views showed to be predominant in the generated equations, but discarding other variables reduced the correlation to 0.7, highlighting that each data entry contributed to the model accuracy. Having an extra *X* variable showed the huge impact of student’s personal factors on any perfect prediction. The nature of the *X* variable should be further investigated through student’s surveys, in order to complete the image of factors determining a student’s academic performance and more advanced learning models should be tested for the proposed predictive model, such as artificial neural networks, Gaussian process regression and ensembles of regression trees.

## Figures and Tables

**Table 1:** List of the generated equations from simple linear regression with or without *X* variable with all variables or using *E* (last page view), *F* (total pages views), *G* (practicals) and *H* (assignments).

Modelling Type	Equation Number	Equation
All Variables Are in Court		
With Outliers		
Simple linear regression model without optimization	(1)	Y=−88+1.28H+0.52G+0.48E+0.05B+0.01C+0.008F−0.004A+0.002D
Simple linear regression with a new predictor (adjusting variable *X*)	(2)	Y=−30.66−5.059X+2.346E+0.69H+0.456G−0.056D−0.034A+0.011C+0.006F+0.003B
Without Outliers		
Simple linear regression model without optimization	(3)	Y=−110.7+1.4H+1.169E−0.88B+0.593G+0.287A−0.064D−0.034C+0.033F
Simple linear regression with a new predictor (adjusting variable *X*)	(4)	Y=−47.3−4.5X+2.2E+0.75H+0.51G−0.5B−0.118D+0.046F−0.036C−0.015A
*E*, *F*, *G* and *H* Variables Are in Court		
With Outliers		
Simple linear regression model without optimization	(5)	Y=−96.7+1.3H+0.8E+0.582G+0.012F
Simple linear regression with a new predictor (adjusting variable *X*)	(6)	Y=−86.92+6.95X+1.268E+1.009G−0.15H+0.008F
Without Outliers		
Simple linear regression model without optimization	(7)	Y=−98.3+1.22H+0.973E+0.56G+0.0217F
Simple linear regression with a new predictor (adjusting variable *X*)	(8)	Y=−100+5X+2.65E+0.68G+0.48H+0.0023F

**Table 2:** Statistical metrics of the generated models, with or without X variable.

Modelling Type	Equation Number	Number of Observation Error Degrees of Freedom	Root Mean Squared Error	Pearson Correlation Coefficient R^2^ Adjusted R^2^	*p*-Value
All Variables Are in Court					
With Outliers					
Simple linear regression model without optimization	(1)	55 46	14.1	0.600 0.350 0.237	7.01 × 10^−3^ **
Simple linear regression with a new predictor (adjusting variable *X*)	(2)	55 45	0.0656	1 1	2.98 × 10^−6^ ***
Without Outliers					
Simple linear regression model without optimization	(3)	47 38	12.4	0.724 0.524 0.424	1.89 × 10^−4^ ***
Simple linear regression with a new predictor (adjusting variable *X*)	(4)	47 37	0.276	1 1	1.77 × 10^−64^ ***
*D*, *E*, *G* and *H* Variables Are in Court					
With Outliers					
Simple linear regression model without optimization	(5)	55 50	13.6	0.585 0.342 0.289	2.72 × 10^−4^ ***
Simple linear regression with a new predictor (adjusting variable *X*)	(6)	55 49	0.264	1 1	3.18 × 10^−87^ ***
Without Outliers					
Simple linear regression model without optimization	(7)	47 42	12.1	0.700 0.493 0.445	7.28 × 10^−6^ ***
Simple linear regression with a new predictor (adjusting variable *X*)	(8)	47 41	0.106	1 1	1.36 × 10^−89^ ***

** and *** are used to indicate *p* value less than 0.01 and 0.001 respectively.

## Data Availability

Not applicable.

## Supplementary Materials

The following are available online at https://www.mdpi.com/article/10.3390/pharmacy10010004/s1, Figure S1: Virtual learning environment engagement indicators and coursework marks that are used in the study as input data for all students (N = 55 students). The *y*-axis represents the value of the indicator (total participation number, number of days since last login and total number of page views) or the coursework and practical marks per student (*x*-axis)., Figure S2: Virtual learning environment engagement indicators and coursework marks that are used in the study as input data for students without outliers (N = 47 students). The *y*-axis represents the value of the indicator (total participation number, number of days since last login and total number of page views) or the coursework and practical marks per student (*x*-axis)., Figure S3: Final exam marks prediction using the developed models versus the original/actual exam marks: (a) with outliers (55 students), (b) without outliers (47 students).
