# Supplementary material for: Remote Learning in Transnational Education: Relationship between Virtual Learning Engagement and Student Academic Performance in BSc Pharmaceutical Biotechnology
Source: Pharmacy (Basel). 2021 Dec 27;10(1):4. doi: 10.3390/pharmacy10010004 (PMC8788569; doi:10.3390/pharmacy10010004)
Supplement: Supplementary file 1 [file pharmacy-10-00004-s001.zip › pharmacy-1489810-supplementary.pdf]

pharmacy

Article

Remote Learning in Transnational Education: Relationship between Virtual Learning Engagement and Student Academic Performance in BSc Pharmaceutical Biotechnology

Taher Hatahet[1,2*], Ahmed A.Raouf Mohamed[3], Maryam Malekigorji[1,2], Emma K. Kerry[1,2]

[1] School of Pharmacy, Queens University Belfast, Belfast BT9 7BL, UK; m.malekigorji@qub.ac.uk (M.M.); E.Kerry@qub.ac.uk (E.K.K.)
[2] China Medical University and Queen's University Joint College, Shenyang, 110122, China
[3] School of Electronics, Electrical Engineering and Computer Science, Queens University Belfast, Belfast BT9 5AG, UK; amohamed06@qub.ac.uk
* Correspondence: t.hatahet@qub.ac.uk

Abstract: The 21st century has seen dramatic changes to education delivery which has widened the scope of transnational education and remote learning via various virtual learning environments (VLEs). Efficient remote teaching activities require students to be engaged with taught materials and academic staff, and for educators to be able to track and improve student engagement. This article describes the generation of a predictive mathematical model for students' exam performance using VLE engagement indicators and coursework marks together to enable the creation of a model with a correlation coefficient of 0.724. This article examines the relationship of each of these variables to final exam marks as well as the addition of personal related variable X on the generated model's accuracy. Generated models show that each variable has a different impact on the prediction of the final exam mark. Results analysis suggests that coursework marks and total VLE page views are the major attributes, while personal factors are also found to greatly impact model accuracy. Considering the case of outliers, who were students with low VLE engagement achieving high exam marks, it is proposed that personal factors, such as behavioural factors and study style, also have a significant effect on student academic attainment. The generated model can be used by students to improve self-efficacy by adjusting their study style and by educators to provide early interventions to support disengaged students. This model can be replicated in different remote learning settings and transnational education, and the findings might be insightful for courses with remote learning strategies to investigate the key educational, personal and engagement parameters for student overall success.

Keywords: Academic performance, linear regression, modelling, optimization, student engagement, transnational education, virtual learning environment

Citation: Lastname, F.; Lastname, F.; Lastname, F. Title. *Pharmacy* **2022**, *10*, 4. https://doi.org/10.3390/pharmacy10010004

Received: 16 November 2021
Accepted: 22 December 2021
Published: 27 December 2021

Publisher's Note: MDPI stays neutral with regard to jurisdictional claims in published maps and institutional affiliations.

Copyright: © 2021 by the authors. Licensee MDPI, Basel, Switzerland. This article is an open access article distributed under the terms and conditions of the Creative Commons Attribution (CC BY) license (https://creativecommons.org/licenses/by/4.0/).

Appendix A: Supplementary data

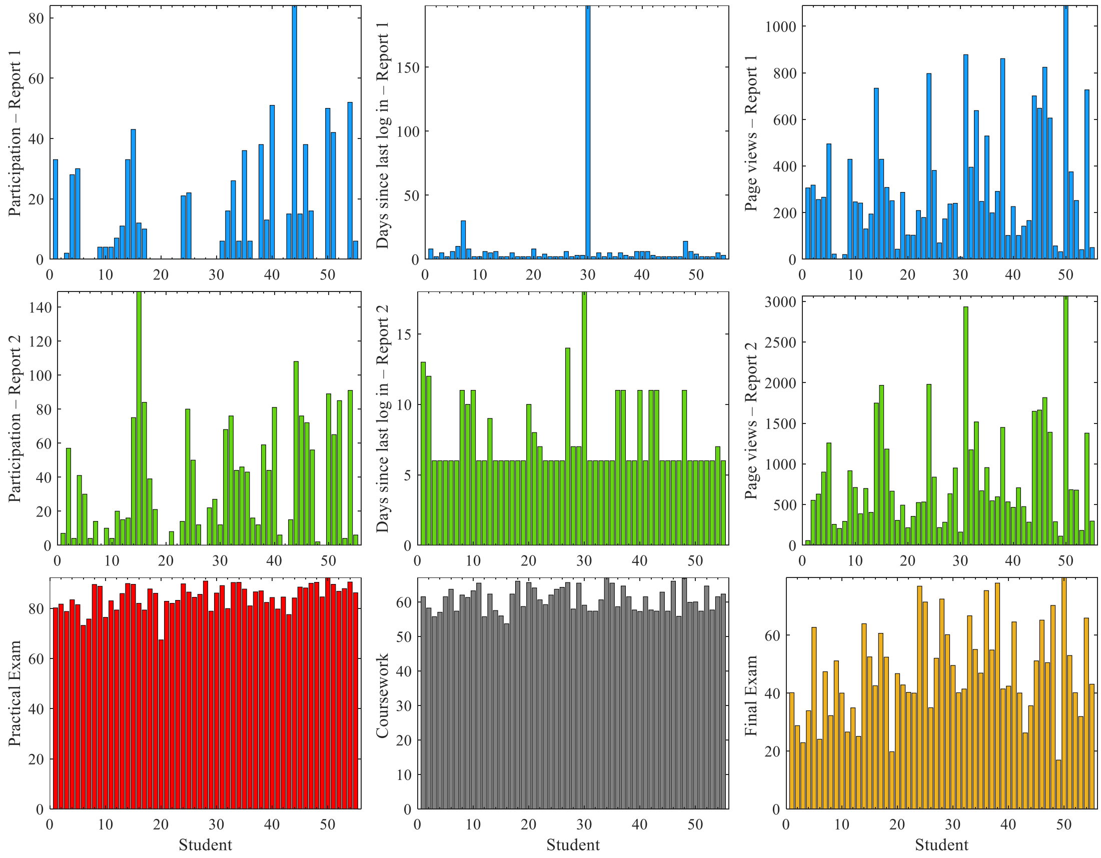

Supplementary Figure S1: Virtual learning environment engagement indicators and coursework marks that are used in the study as input data for all students (N=55 students). The *y*-axis represents the value of the indicator (total participation number, number of days since last login and total number of page views) or the coursework and practical marks per student (x-axis).

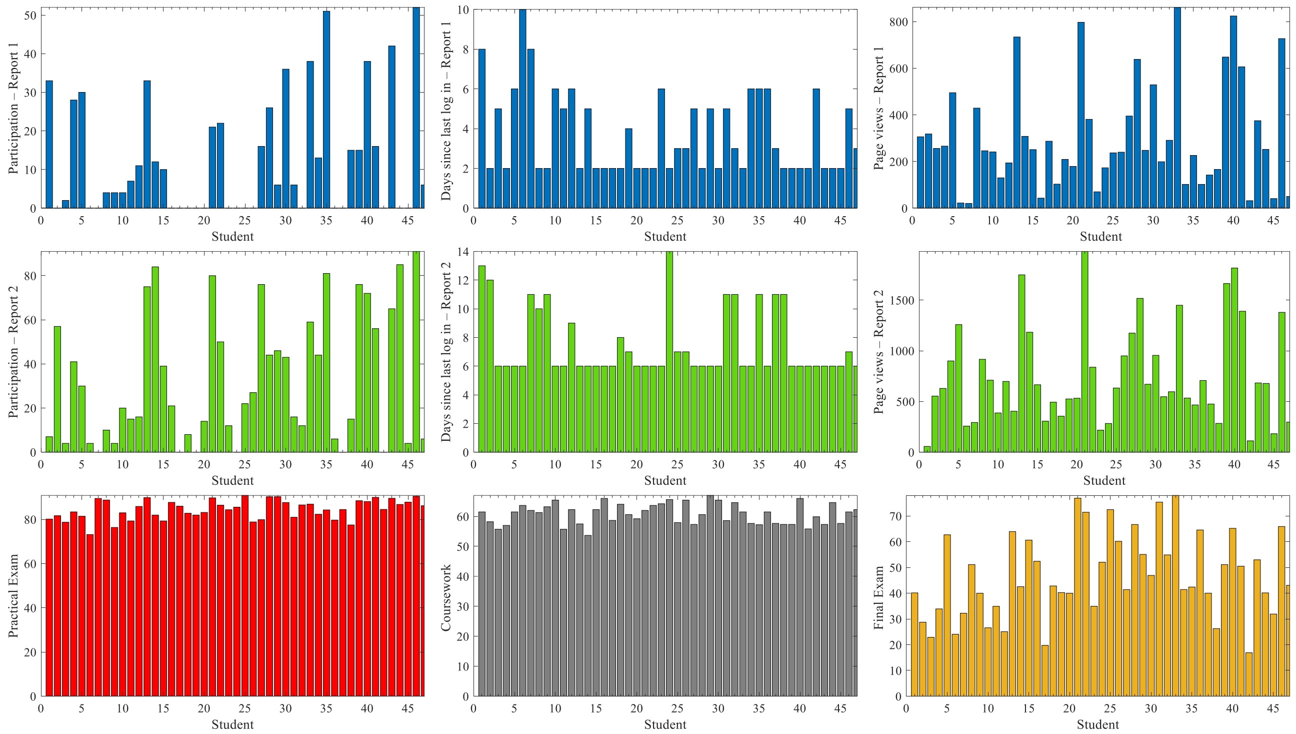

Supplementary Figure 2: Virtual learning environment engagement indicators and coursework marks that are used in the study as input data for students without outliers (N=47 students). The *y*-axis represents the value of the indicator (total participation number, number of days since last login and total number of page views) or the coursework and practical marks per student (x-axis).

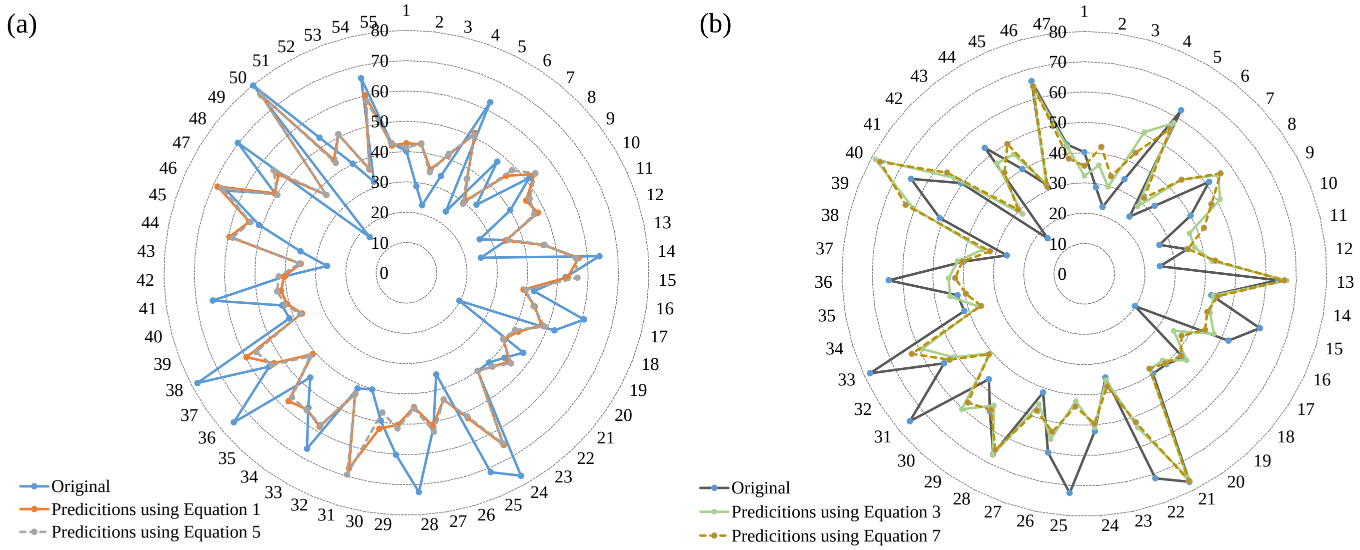

Supplementary Figure 3: Final exam marks prediction using the developed models versus the original/actual exam marks: (a) with outliers (55 students), (b) without outliers (47 students)
